# Heterogeneous profiles of self-directed learning competencies among post-2000s Chinese nursing students: a latent profile analysis and associated factors

**DOI:** 10.1186/s12912-026-05183-w

**Published:** 2026-08-07

**Authors:** Zuyao Xiao, Ruiqing Cui, Jiaxin Liu, Guohua Jing, Lingyun Wei, Mina Huang, Ying Liu, Tao Liu

**Affiliations:** 1 School of Nursing, Jinzhou Medical University, 40 Songpo Road, Linghe, Jinzhou, Liaoning 121001 China; 2 Pharmacy School, Jinzhou Medical University, 40 Songpo Road, Linghe, Jinzhou, Liaoning 121001 China

**Keywords:** Self-directed learning, Latent profile analysis, Post-2000s generation, Nursing education

## Abstract

**Background:**

The nursing profession requires practitioners to possess the ability to engage in lifelong learning. As the core of the future nursing workforce, the self-directed learning abilities of nursing students born in the 2000s are of critical importance. This study aims to identify the characteristics of self-directed learning abilities and their influencing factors among different categories of nursing students through latent profile analysis, thereby providing a basis for developing targeted intervention strategies.

**Methods:**

In April 2026, a cross-sectional survey was conducted using a structured questionnaire among nursing students at two medical universities in Liaoning and Shandong provinces. Latent profile analysis was used to identify latent categories of self-directed learning ability among nursing students born in the 2000s; independent samples t-tests, one-way ANOVA, or corresponding nonparametric tests (Mann-Whitney U test, Kruskal-Wallis test) were employed for intergroup comparisons; variables found to be statistically significant in the univariate analysis were included in an ordered logistic regression model to analyze the factors influencing the categories.

**Results:**

The self-directed learning abilities of nursing students born in the 2000s can be classified into three latent categories: the “Comprehensive Weakness Group” (18%), the “Balanced Development Group” (48%), and the “Comprehensive Leadership Group” (34%). Ordered logistic regression analysis revealed that educational background, whether serving as a student leader or class committee member, future plans to pursue a career in nursing, prior training in self-directed learning, academic burnout, and learning motivation were all significantly associated with the category of self-directed learning ability (all *P* < 0.05).

**Conclusion:**

Differentiated educational interventions should be implemented for nursing students with different characteristics. For the “Comprehensive Weakness Group,” psychological support and training in structured self-directed learning skills should be strengthened; the “Balanced Development Group” should be provided with appropriate guidance to facilitate their transition to the “Comprehensive Leadership Group,” and the exemplary role of the “Comprehensive Leadership Group” should be fully leveraged to enhance the self-directed learning abilities of all post-2000s nursing students.

**Clinical trial number:**

Not applicable.

**Trial registration:**

Not applicable.

## Background

The accelerating global aging of the population and rapid innovations in medical technology are placing increasingly high demands on nursing professionals, who must continuously update their knowledge and skills to deliver safe, high-quality care in dynamic clinical environments [1–3]. Self-directed learning (SDL)—the capacity to independently assess learning needs, set goals, select strategies, and evaluate outcomes—represents a core generic competency that underpins effective nursing practice across diverse professional contexts [4]. Unlike discipline-specific skills, SDL competencies are broadly transferable and particularly vital in nursing, a profession that explicitly requires lifelong learning [5]. As emerging technologies such as artificial intelligence penetrate the healthcare sector with increasing speed, cultivating SDL among nursing students has become an educational imperative rather than an optional aspiration [6, 7]. Post-2000s nursing students, who will constitute the future backbone of the profession, are the target population in which this cultivation must begin [8, 9]. 

Evidence consistently demonstrates that nursing students with stronger SDL competencies perform better academically, regulate their learning more effectively, adapt more readily to new environments, and develop stronger critical thinking and clinical decision-making skills [10–17]. In clinical practice, nurses with well-developed SDL abilities can keep pace with evolving evidence, thereby enhancing patient care quality and professional self-efficacy [11]. Despite these established benefits, surveys from China, Australia, Iran, and other countries consistently report that nursing students’ SDL remains at only a moderate level, and some students perceive learning as an additional burden rather than a professional responsibility [18]. This persistent gap between the high demands of nursing practice and the current SDL competency level underscores the urgency of understanding the characteristics and determinants of SDL among post-2000s Chinese nursing students.

Although a body of literature has examined factors associated with SDL in nursing students, most prior studies have employed variable-centered approaches—such as correlation analysis or multiple regression—which treat the sample as a single homogeneous group and yield estimates of average effects across all participants. Such methods are inherently limited in their ability to detect meaningful subgroup variation: they cannot reveal whether distinct subpopulations with qualitatively or quantitatively different SDL profiles exist, nor can they identify which individuals belong to a particularly vulnerable or high-performing subgroup that may require differentiated educational attention. Critically, a regression model can identify that “academic burnout is negatively associated with SDL on average.” However, it cannot determine whether a subset of students simultaneously experiences high burnout and low SDL across all dimensions. This pattern would call for targeted institutional support rather than generalized curriculum adjustments.

Latent profile analysis (LPA) is a person-centered statistical method specifically designed to address this theoretical gap. Rather than estimating coefficients for the average student, LPA probabilistically classifies individuals into latent subgroups based on their multivariate response patterns, thereby revealing the heterogeneous structure of SDL within the population [19, 20]. This approach is educationally meaningful for at least three reasons. First, identification of distinct SDL profiles enables the design of profile-specific interventions rather than a one-size-fits-all approach; students in a “low across all dimensions” profile require fundamentally different support from those who are “high in motivation but low in self-monitoring.” Second, understanding the prevalence of each profile allows institutions to allocate pedagogical resources efficiently, prioritizing the most vulnerable subgroup. Third, by incorporating SDL training history, academic burnout, and motivation simultaneously as profile correlates, LPA can map the ecological context of each subgroup in a way that regression coefficients for individual variables cannot capture. LPA has been increasingly applied in nursing education research to characterize heterogeneity in professional identity, patient safety competency, professional calling, and resilience [21, 22]. However, to date, no published study has applied this person-centered framework specifically to SDL competencies among post-2000s Chinese nursing students—a cohort with distinctive generational characteristics, educational experiences, and career pressures that may produce SDL profiles not captured in earlier research.

Therefore, this study aimed to: (1) identify latent heterogeneous profiles of SDL competencies among post-2000s Chinese nursing students using LPA; (2) compare demographic characteristics, academic burnout, and learning motivation across profiles; and (3) explore independent predictors of profile membership. The findings are intended to provide empirical evidence for nursing educators seeking to implement stratified, evidence-based educational interventions that promote SDL development across diverse student subgroups.

## Methods

### Research design

This study is a cross-sectional study conducted in accordance with the STROBE guidelines.

### Recruitment of participants

Participants were recruited using convenience sampling from two medical universities in Liaoning Province and Shandong Province, China, in April 2026. Researchers contacted class advisors at each institution, who distributed the electronic questionnaire link via WeChat to all students enrolled in nursing programs who met the eligibility criteria. All eligible students in each participating class were invited to participate; participation was entirely voluntary. A total of 430 students were identified as eligible and received the questionnaire invitation. Of these, 410 returned completed questionnaires (non-response rate: 4.7%), and 12 were subsequently excluded for failing quality control checks, yielding 398 valid responses (valid response rate: 92.6%).

Inclusion criteria: (1) Full-time nursing students currently enrolled at the participating institutions; (2) born in or after 2000, fulfilling the generational definition of “post-2000s”; (3) willing to participate voluntarily and providing written informed consent.

Exclusion criteria: (1) Students temporarily absent from school due to sick leave, leave of absence, suspension of studies, or other reasons; (2) students unwilling to participate.

### Sample size calculation

No standardized formula exists for LPA-specific sample size calculation. Simulation studies have demonstrated that when scale reliability is high (Cronbach’s α > 0.90) and class separation is adequate, samples of 300–500 provide stable LPA solutions [30]. Based on this guidance, a minimum of 360 valid responses was targeted. Accounting for an anticipated invalid questionnaire rate of approximately 10%, a minimum of 400 questionnaires needed to be distributed (360 ÷ 0.90 ≈ 400). Accordingly, 430 questionnaires were distributed. After quality control, 398 valid responses were retained, exceeding the pre-specified minimum [23]. 

Questionnaires were excluded as invalid if completed in less than 120 s. This threshold was established based on the estimated minimum reading time for the complete instrument: the questionnaire contains approximately 800 Chinese characters across all scale items; at a typical reading speed of 400–500 characters per minute, conscientious completion requires at least 100–120 s. Questionnaires completed below this threshold were therefore considered to reflect insufficient engagement with the survey content.

### Instruments

#### General demographic questionnaire

For this study, a general demographic questionnaire was designed. It included age, gender, educational background, GPA (top 30% or bottom 70%), place of residence (urban or rural), daily mobile phone usage (less than 6 h or more than 6 h), experience as a student council or class committee member, plans to pursue a career in nursing, and whether the respondent had received training related to self-directed learning skills.

#### Self-Directed Learning Ability Inventory (SDLI)

The original scale was developed by Cheng et al. and consists of 20 items across four dimensions: learning motivation (6 items), planning and implementation (6 items), self-monitoring (4 items), and interpersonal communication (4 items) [18]. After Guo et al. conducted cross-cultural adaptation and reliability and validity tests on the Chinese version of the scale, exploratory factor analysis led to adjustments in item allocation. The final Chinese version retained 20 items across four dimensions: learning motivation (Q1–Q6, 6 items), planning and implementation (Q7–Q11, 5 items), Self-Monitoring (Q12–Q16, 5 items), and Interpersonal Communication (Q17–Q20, 4 items) [24]. All items are evaluated on a 5-point Likert scale (ranging from 1 = “strongly disagree” to 5 = “strongly agree”). The total score ranges from 20 to 100, with a higher score indicating a stronger self-directed learning ability. After validation of the translation, the Cronbach’s alpha coefficient for the Chinese version of the SDLI was 0.916. In this study, the Cronbach’s alpha coefficient for this scale was 0.933.

#### Academic burnout scale

The third scale used was the College Student Academic Burnout Scale, developed by Lian Rong et al. This scale consists of 20 items across three dimensions: low mood (8 items), low sense of accomplishment (6 items), and maladaptive behavior (6 items). Items 1, 3, 6, 8, 11, 13, 15, and 18 are reverse-scored [25]. The total score ranges from 20 to 100, with a higher score indicating a more severe level of academic burnout. In this study, the Cronbach’s alpha coefficient for this scale was 0.912, indicating good internal consistency.

#### Learning motivation scale

The Learning Motivation Scale is based on a questionnaire adapted and localized by Chi Liping et al. from the scale developed by Amabile et al. It consists of 16 items across two dimensions: intrinsic motivation (8 items) and extrinsic motivation (8 items). All items are scored on a positive scale [26]. A higher score indicates stronger motivation to learn. In this study, the Cronbach’s alpha coefficient was again measured at 0.907, indicating good internal consistency.

### Survey collection

After obtaining approval from the school and support from teachers, the researchers created an electronic questionnaire using the Questionnaire Star platform. With the assistance of teachers, they explained the purpose and content of the study to potential participants in advance and obtained their informed consent. The questionnaire link was sent via WeChat. Prior to the formal survey, a pilot test was conducted with 10 students to assess item clarity. As no revisions were deemed necessary following pilot testing, these 10 students were included in the formal analysis (constituting part of the final 398 valid responses). To ensure data quality, this study implemented two key quality control measures: (1) IP restrictions were enabled to prevent duplicate responses from the same participant; (2) all questions were marked as mandatory, and the questionnaire could only be submitted after all questions were completed, thereby avoiding missing values.

Missing data. As described in Sect.  2.5, all questionnaire items were designated as mandatory, and the online platform prevented submission of incomplete responses. Consequently, no item-level missing values were present in the dataset (0% missing rate across all 398 participants and all variables). The absence of missing data was verified prior to analysis by inspecting the Mplus data summary output, which confirmed complete data for all 20 SDLI indicators and all covariate variables. Because no missing data existed, no imputation procedures were required. The potential for systematic dropout to have introduced selection bias—for example, if students with the lowest SDL competencies were disproportionately likely to abandon the survey—cannot be fully excluded; however, the platform-enforced mandatory completion requirement eliminated item-level nonresponse within submitted questionnaires, and the overall nonresponse rate of 4.7% (20 students who received the link but did not submit a questionnaire) was low and unlikely to have materially distorted the profile structure.

### Data analysis

#### Latent Profile Analysis (LPA)

LPA was conducted in Mplus 8.3, using scores on all 20 individual SDLI items as continuous indicators. Item-level rather than dimension-level scores were used as indicators for three reasons: (1) item-level analysis preserves fine-grained variation within each SDL dimension, avoiding masking effects that can arise when individual items are aggregated into subscale totals; [27] (2) item-level LPA indicators have been successfully applied in prior nursing LPA studies, demonstrating advantages in identifying more refined subgroup structures; [33, 34] and (3) the sample-to-indicator ratio of approximately 20:1 (398 observations per 20 indicators) meets established empirical guidelines [28]. 

##### Model specification

All LPA models were estimated under the default variance-constrained specification in Mplus, whereby within-class item variances were constrained to be equal across latent profiles (i.e., homoscedastic specification). This specification was selected over the freely estimated variance model for two reasons: (1) it substantially improves model convergence and solution stability, which is particularly important when the number of indicators is large relative to sample size; and (2) it is the predominant specification in applied LPA research within the nursing and health sciences literature, facilitating comparability of results [29, 30]. The local independence assumption—which specifies that all inter-item covariances within each latent class are fixed to zero—was accepted without modification, consistent with standard LPA practice. This assumption posits that the latent class variable fully accounts for the observed associations among indicators, such that items are uncorrelated within classes. Given the factorial structure of the SDLI (items loading on four theoretically distinct subscales), residual within-class covariances among items of the same subscale cannot be fully excluded; however, given the high entropy value (0.953) and well-separated profile structure obtained in the final solution, the impact of any residual within-class covariance on class enumeration is expected to be minimal [23]. 

##### Estimator

All models were estimated using the maximum likelihood estimator with robust standard errors (MLR) as implemented in Mplus 8.3. MLR provides robust parameter estimates under conditions of non-normality of continuous indicators, which is appropriate for Likert-type item responses [31]. Model enumeration and selection. To guard against local maxima solutions, each model was estimated using 200 sets of random starting values with 50 final-stage optimizations, and the stability of the final log-likelihood was verified across multiple replications. Models ranging from 1 to 5 classes were evaluated sequentially. Model selection was guided by the Akaike Information Criterion (AIC), Bayesian Information Criterion (BIC), and sample-size-adjusted BIC (aBIC), for which lower values indicate better fit [32, 33]. The Lo-Mendell-Rubin (LMR) adjusted likelihood ratio test and the Bootstrap Likelihood Ratio Test (BLRT) were used to evaluate whether the k-class model significantly improved fit over the k–1 class model (*P* < 0.05). Classification accuracy was assessed using entropy, with values > 0.80 indicating adequate class separation.

Minimum class size criterion. As an additional substantive criterion for model selection, each retained latent class was required to contain a minimum of 5% of the total analytical sample (*n* ≥ 20), consistent with recommendations in the applied LPA literature that guard against the extraction of numerically trivial classes that may represent estimation artifacts or extreme outliers rather than theoretically meaningful subgroups. This criterion was applied alongside the statistical fit indices to adjudicate between competing models: if a higher-class solution yielded superior statistical fit but contained one or more classes below the 5% threshold, the more parsimonious solution was preferred unless substantive theoretical justification could be provided for the small class. As reported in Sect.  3.2, the smallest class in the retained 3-class solution comprised 18.0% of the sample (*n* = 71), well above this threshold, whereas the 5-class solution produced one class comprising approximately 6% of the sample (*n* ≈ 24), contributing to the decision to prefer the 3-class solution.

#### Analysis of influencing factors

All secondary analyses were performed in IBM SPSS Statistics 29. For descriptive statistics, categorical variables were reported as frequencies and percentages; normally distributed continuous variables as mean ± SD; and non-normally distributed continuous variables as median (interquartile range, IQR). Normality of continuous variables (age, academic burnout score, and learning motivation score) was assessed using the Shapiro-Wilk test prior to group comparisons. All three variables departed significantly from normality (all Shapiro-Wilk *P* < 0.05); accordingly, between-group comparisons for these variables were conducted using the non-parametric Kruskal-Wallis H test, with pairwise post-hoc comparisons performed via Mann-Whitney U test with Bonferroni correction for multiple comparisons. One-way ANOVA was not applied to these variables. Descriptive statistics for non-normally distributed continuous variables are reported as median (interquartile range, IQR) throughout. For SDL dimension scores (Table 3), Levene’s test was used to assess homogeneity of variances; Tukey’s HSD post-hoc test was applied to dimensions with equal variances, and the Games-Howell post-hoc test to dimensions with unequal variances. Categorical variables were compared using chi-square tests.

Variables that achieved statistical significance in univariate analyses (*P* < 0.05) were entered simultaneously into an ordinal logistic regression model. This data-driven variable selection strategy was adopted because no mature theory-based framework currently specifies an exhaustive set of SDL predictors applicable to the post-2000s Chinese nursing student population; the exploratory univariate screening approach minimizes the risk of model overfitting given the moderate sample size (*n* = 398) while ensuring that only empirically relevant variables are retained.

Justification for ordinal logistic regression. The choice between ordinal and multinomial logistic regression was determined through two complementary evaluations conducted prior to the regression analysis.

First, the proportional odds assumption was formally evaluated using the parallel lines test; satisfaction of this assumption (χ² = 5.391, df = 7, *P* = 0.612) statistically confirmed that a single set of regression coefficients adequately describes the relationship between predictors and the outcome across all response thresholds, thereby supporting the use of ordinal logistic regression.

**Fig. 1:**
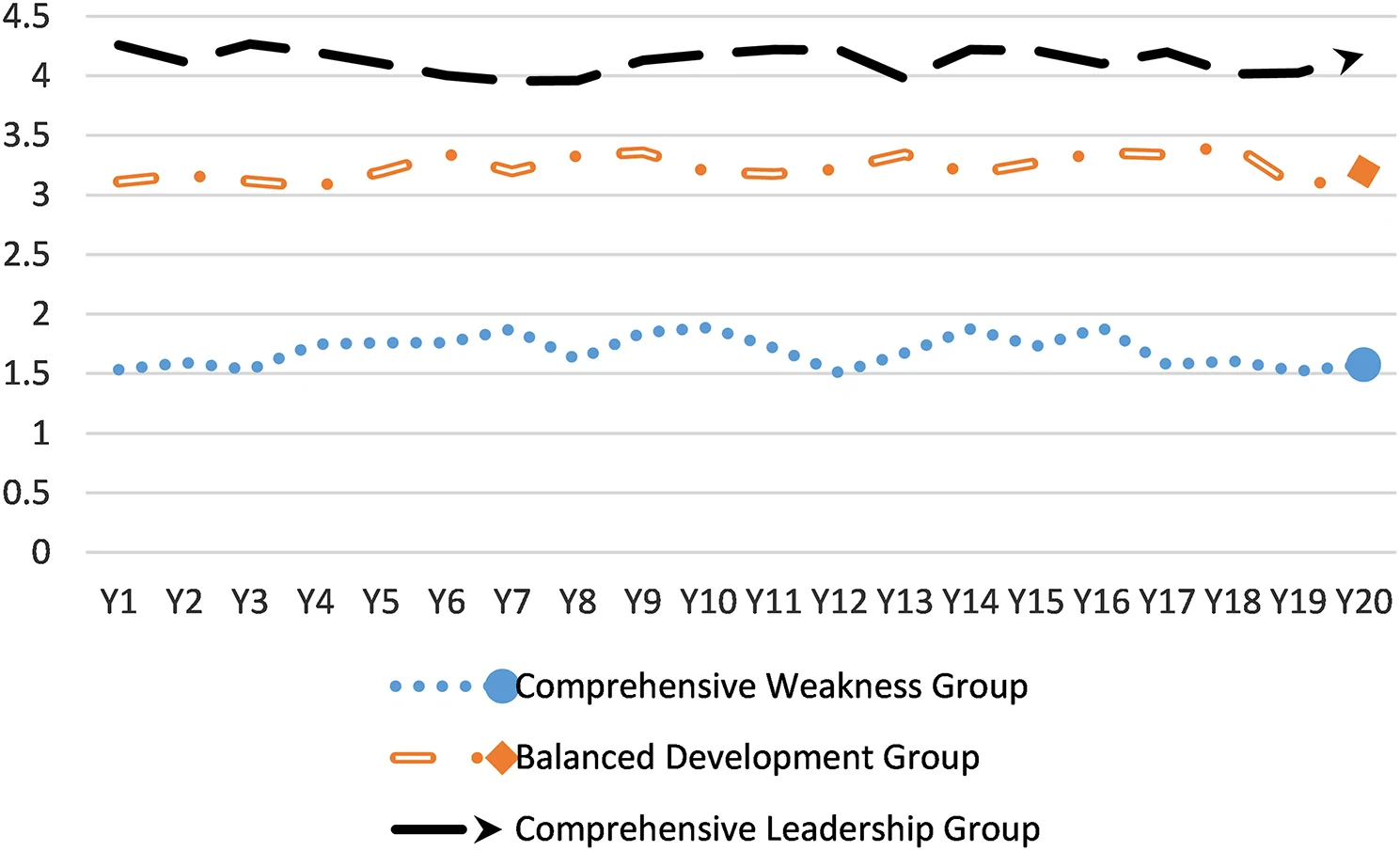
Item-level SDL score profiles across three latent classes

Second, the ordinal nature of the three latent profiles was empirically verified through inspection of the item-level and dimension-level SDL score patterns. Specifically, we examined whether the three profiles differed primarily in score magnitude (level differences, consistent with an ordinal hierarchy) or in the relative ranking of SDL dimensions across profiles (shape differences, which would indicate qualitatively distinct configurations and mandate multinomial logistic regression). As reported in Sect.  3.2, Table 3, and Fig. 1, all four SDL dimensions maintained a consistent rank ordering across the three classes (Class 1 < Class 2 < Class 3) with no crossing or reversals: mean dimension scores ranged from 9.93 to 25.02 (learning motivation), 8.94–20.47 (planning and implementation), 8.68–20.77 (self-monitoring), and 6.28–16.39 (interpersonal communication), all exhibiting a monotonically increasing gradient from the Comprehensive Weakness Group to the Comprehensive Leadership Group. No class ranked higher on any one SDL dimension while ranking lower on another—the defining criterion for shape differences. The item-level profiles displayed in Fig. 1 corroborate this pattern: all 20 item trajectories are broadly parallel across the three latent classes, reflecting systematic differences in overall SDL level rather than qualitatively distinct configurational patterns.

The convergence of these two lines of evidence—statistical (parallel lines test) and descriptive (profile shape inspection)—jointly justifies the use of ordinal logistic regression as the appropriate modeling choice for predicting SDL profile membership in this sample.

Methodological note on the classify-analyze approach. In the present study, latent class memberships were derived via modal posterior assignment in Mplus and subsequently transferred to SPSS for ordinal logistic regression—an approach commonly referred to as “classify-analyze.” We acknowledge that this approach has been critiqued in the mixture modeling literature for potentially underestimating standard errors and inflating effect sizes when classification uncertainty is non-trivial. However, three conditions present in this study substantially mitigate these concerns. First, the 3-class model achieved an entropy of 0.953, indicating that approximately 95.3% of the total classification uncertainty is resolved; simulation research has demonstrated that when entropy exceeds 0.80, modal assignment introduces negligible bias in subsequent regression estimates. Second, the average posterior probabilities of correct class assignment for the three classes were 0.97, 0.96, and 0.95, respectively, all substantially exceeding the widely accepted threshold of 0.70, confirming that class boundaries are sharp and that modal assignment closely approximates the true latent class structure. Third, the three profiles differed primarily in overall score level rather than in profile shape (see Sect.  3.2 and Fig. 1), which further reduces the risk of cross-class misclassification influencing regression coefficients in a directionally inconsistent manner. Under these conditions, the classify-analyze approach produces results closely approximating those of modern auxiliary variable methods such as BCH or R3STEP, as has been verified in simulation studies. The findings of this study should nonetheless be interpreted with the understanding that this is an exploratory, cross-sectional analysis, and replication using integrated three-step approaches is encouraged in future research.

All tests were two-sided with a significance level set at α = 0.05.

### Ethical considerations

This study was approved by the Research Ethics Committee of Jinzhou Medical University (Approval No.: JZMULL2026193) and conducted in accordance with the principles of the Declaration of Helsinki. All participants received a full explanation of the study’s purpose and procedures prior to data collection and provided written informed consent before participation. Participation was entirely voluntary; participants retained the right to withdraw at any time without obligation to provide a reason. All personal data were handled in strict confidence, and the collected information was used exclusively for the academic purposes of this study.

## Results

### Demographic characteristics

After excluding invalid questionnaires, this study ultimately included 398 participants with mean age of 22.05 ± 2.06 years; among them, 313 were female (78.6%) and 85 were male (21.4%); In terms of educational level, 230 participants (57.8%) were undergraduates, and 168 (42.2%) were graduate students. The total SDL scale score was 65.29 ± 17.45 points (mean ± SD), the total academic burnout score was 73.25 ± 10.54 points, and the total learning motivation score was 62.74 ± 8.56 points. Detailed general demographic data and the distribution of scores for each scale are presented in Table 1.

**Table 1:** Demographic characteristics

Variables	Categories	Mean(SD)	Frequency(*N*)	Percentage
Age		22.05 ± 2.06		
Gender	Female		313	78.60
	Male		85	21.40
Place of residence	Urban		230	57.80
	Rural		168	42.20
GPA	Top 30%		166	41.70
	Bottom 70%		232	58.30
Daily mobile phone usage time	< 6 h		189	47.50
	> 6 h		209	52.50
Current Enrollment	Bachelor’s		230	57.80
	Master’s		168	42.20
Experience as a student council or class committee member	Yes		193	48.50
	No		205	51.50
Interest in pursuing a career in nursing	Yes		281	70.60
	No		117	29.40
Experience with training related to self-directed learning	Yes		125	31.40
	No		273	68.60
SDL Scale		65.29 ± 17.45		
Academic Burnout Scale		73.25 ± 10.54		
Learning Motivation Scale		62.74 ± 8.56		

Note: Continuous variables are presented as mean ± SD; categorical variables as frequency (N) and percentage (%). The “%” symbol is omitted from data cells and indicated in the column header

### Characteristics of self-directed learning abilities among students in different categories

LPA models with 1 to 5 classes were estimated. Fit statistics are presented in Table 2. AIC, BIC, and aBIC decreased consistently as the number of classes increased; however, BIC increased slightly when moving from the 3-class to the 4-class model, indicating diminishing returns in model fit. The 3-class model achieved an entropy of 0.953, reflecting excellent classification accuracy. The LMR test demonstrated that the 3-class model represented a statistically significant improvement over the 2-class model (*P* < 0.001), whereas the 4-class model did not significantly improve upon the 3-class solution (*P* = 0.399), and the 5-class model’s LMR test was similarly non-significant (*P* = 0.187). Regarding minimum class size, the smallest class in the 3-class solution contained 18.0% of the total sample (*n* = 71), well above the 5% threshold. In the 5-class solution, one class contained only 6% of the sample (*n* ≈ 24), raising concerns about stability. Taken together, the statistical indices and substantive interpretability criteria supported the 3-class model as the optimal solution.

Based on the SDL performance characteristics of each class, Class 1 was designated the “Comprehensive Weakness Group” (18.0%, *n* = 71), Class 2 the “Balanced Development Group” (48.0%, *n* = 191), and Class 3 the “Comprehensive Leadership Group” (34.0%, *n* = 136).

**Table 2:** Model fit

Model	LL	AIC	BIC	aBIC	Entropy	LMR *P*-value	BLRT *P*-value	Category probability
Class1	-13436.97	26953.944	27113.402	26986.48				
Class2	-12071.83	24265.667	24508.84	24315.285	0.998	*P* < 0.001	*P* < 0.001	0.18/0.82
Class3	-11617.13	23398.26	23725.149	23464.959	0.953	*P* < 0.001	*P* < 0.001	0.18/0.48/0.34
Class4	-11565.95	23337.896	23748.5	23421.677	0.91	0.3986	*P* < 0.001	0.18/0.15/0.34/0.33
Class5	-11501.89	23251.785	23746.105	23352.648	0.924	0.1874	*P* < 0.001	0.18/0.15/0.32/0.06/0.29

Note: The 1-class model serves as the unconditional baseline; because there is no k–1 model for comparison, Entropy, LMR, and BLRT values are not applicable and are left blank (—). “Category probability” represents the estimated proportion of the total analytical sample (*N* = 398) probabilistically assigned to each latent class based on modal posterior class assignment in Mplus; proportions are presented in class order (Class 1 / Class 2 / … / Class k). LL = log-likelihood; AIC = Akaike Information Criterion; BIC = Bayesian Information Criterion; aBIC = sample-size-adjusted BIC; LMR = Lo-Mendell-Rubin adjusted likelihood ratio test; BLRT = Bootstrap Likelihood Ratio Test

An important observation regarding the nature of these profiles warrants explicit discussion. As shown in Fig. 1, the three classes exhibit broadly parallel response profiles across all 20 SDLI items: the relative pattern of item scores (i.e., profile shape) is similar across groups, with differences manifesting primarily as systematic variation in score magnitude (i.e., profile level). This indicates that the three profiles represent a quantitative gradient of SDL competency rather than qualitatively distinct configurations of SDL strengths and weaknesses. The profile labels therefore reflect overall SDL proficiency levels rather than distinct SDL archetypes. This quantitative gradient structure provides direct theoretical justification for employing ordinal rather than multinomial logistic regression in the subsequent analysis.

Post-hoc comparisons were conducted following assessment of variance homogeneity via Levene’s test. Tukey’s HSD was applied to dimensions with equal variances (learning motivation, interpersonal communication, and total SDL score; all Levene’s *P* > 0.05); Games-Howell was used for dimensions with unequal variances (planning and implementation, self-monitoring; Levene’s *P* < 0.05). All pairwise comparisons across the three classes were statistically significant (*P* < 0.001); detailed results appear in Table 3.

**Table 3:** Comparison of SDL dimension scores and total SDL score across latent profiles

Variables	Class1 Mean ± SD(*n* = 71)	Class2 Mean ± SD(*n* = 191)	Class3 Mean ± SD(*n* = 136)	F/Welch’s F	*P*
Learning motivation(6 items)	9.93 ± 2.301	18.97 ± 2.686	25.02 ± 2.383	842.526	*P* < 0.001
Planning and implementation(5 items)	8.94 ± 2.484	16.25 ± 3.014	20.47 ± 2.251	431.270	*P* < 0.001
Self-Monitoring(5 items)	8.68 ± 2.316	16.35 ± 2.696	20.77 ± 2.167	665.460	*P* < 0.001
Interpersonal Communication(4 items)	6.28 ± 1.683	13.04 ± 1.946	16.39 ± 1.933	763.830	*P* < 0.001
Total score of the SDL scale(20 items)	33.83 ± 4.876	64.61 ± 5.132	82.65 ± 4.683	2284.350	*P* < 0.001

Note: Variance homogeneity was assessed using Levene’s test prior to post-hoc comparisons. For dimensions with equal variances (learning motivation and interpersonal communication; Levene’s *P* > 0.05), standard one-way ANOVA with Tukey’s HSD post-hoc test was applied. For dimensions with unequal variances (planning and implementation and self-monitoring; Levene’s *P* < 0.05), Welch’s ANOVA with Games-Howell post-hoc test was applied (marked with ᵃ). The total SDL score was analyzed using standard ANOVA with Tukey’s HSD (Levene’s *P* > 0.05). All pairwise comparisons between the three latent classes were statistically significant at *P* < 0.001

### Analysis of demographic characteristics

The results of the univariate analysis indicate that age, educational attainment, whether the student serves as a student council or class committee member, future plans to pursue a career in nursing, whether the student has received training in self-directed learning skills, academic burnout, and learning motivation all influence the three potential profile classifications, with all differences being statistically significant (*P* < 0.05). For details, see Table 4.

**Table 4:** Univariate analysis

Variables	Categories	Class1	Class2	Class3	H/χ2	*P*
Age, M(P25, P75)		21(20,23)	22(20,24)	23(21, 24)	14.21	***P*** < **0.01**
Gender	Female	56	148	109	0.337	0.845
	Male	15	43	27		
Place of residence	Urban	42	114	74	0.972	0.615
	Rural	29	77	62		
GPA	Top 30%	32	79	55	0.429	0.807
	Bottom 70%	39	112	81		
Daily mobile phone usage time	< 6 h	36	92	61	0.709	0.702
	> 6 h	35	99	75		
Current Enrollment	Bachelor’s	64	140	26	132.675	***P*** < **0.01**
	Master’s	7	51	110		
Experience as a student council or class committee member	Yes	13	75	105	77.295	***P*** < **0.01**
	No	58	116	31		
Interest in pursuing a career in nursing	Yes	31	127	123	51.172	***P*** < **0.01**
	No	40	64	13		
Experience with training related to self-directed learning	Yes	4	42	79	74.696	***P*** < **0.01**
	No	67	149	57		
Academic Burnout Scale, M(P25, P75)		84(78,90)	75(68,81)	66(61,69)	162.58	***P*** <** 0.01**
Learning Motivation Scale, M(P25, P75)		57(52,64)	62(57,67)	67(60,71)	38.3	***P*** < **0.01**

Table 4 note: Continuous variables (age, academic burnout, learning motivation) were reported as median (interquartile range, IQR) and compared using the Kruskal-Wallis H test. Pairwise comparisons were conducted using Mann-Whitney U test with Bonferroni correction for multiple comparisons. Categorical variables were presented as frequencies and percentages and compared using chi-square tests. H denotes Kruskal-Wallis test statistic; χ² denotes chi-square test statistic. All tests were two-sided with significance level set at *P* < 0.05

### Test for parallel lines and logistic regression analysis

We conducted a parallel lines test on the significant variables identified in the univariate analysis. The results showed that *P* > 0.05, indicating that the proportional odds assumption was satisfied and that ordered logistic regression analysis could be performed (Table 5). For ordinal logistic regression, category membership was coded as: 1 = “Comprehensive Weakness Group,” 2 = “Balanced Development Group,” 3 = “Comprehensive Leadership Group.” Thus, an OR > 1 indicates a higher likelihood of belonging to a more advanced SDL category, while an OR < 1 indicates a higher likelihood of remaining in a lower category.

**Fig. 2:**
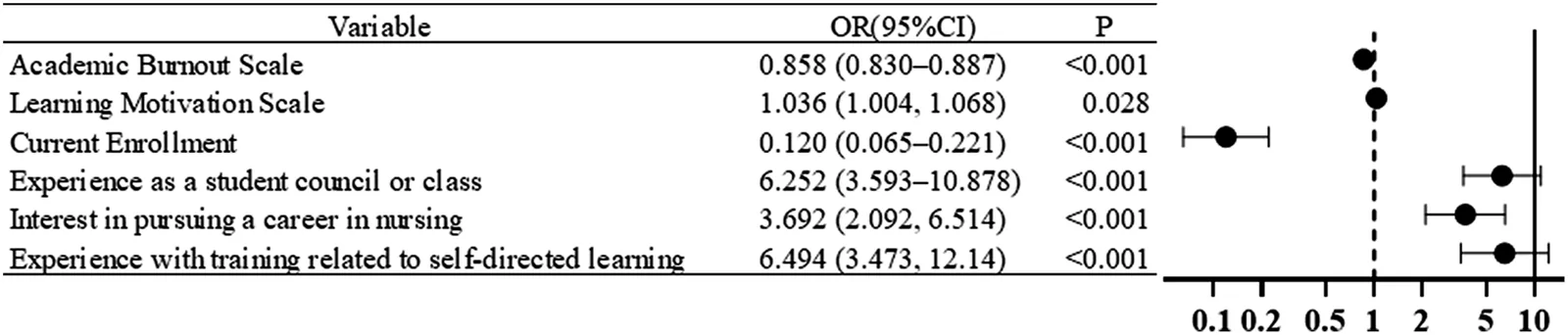
Forest plot of independent predictors of SDL competency level

The results indicated that academic burnout was a barrier to reaching higher levels of SDL ability (B = − 0.153, *P* < 0.001, OR = 0.858, 95% CI: 0.830–0.887). Undergraduates were significantly less likely to reach a higher category compared to master’s students (OR = 0.120, 95% CI: 0.066–0.221). Conversely, serving as a student council or class committee member (OR = 6.252, 95% CI: 3.592–10.877), intending to pursue a career in nursing (OR = 3.692, 95% CI: 2.092–6.514), having received training related to self-directed learning skills (OR = 6.494, 95% CI: 3.473–12.147), and learning motivation (OR = 1.036, 95% CI: 1.004–1.068, *P* = 0.028) were factors promoting membership in higher SDL categories (all *P* < 0.05). See Table 6 for details and Fig. 2 for the logistic regression forest plot.

The predictive role of age was not statistically significant in the multivariable model (B = − 0.007, *P* = 0.907, OR = 0.993, 95% CI: 0.878–1.122). Multicollinearity diagnostics confirmed that this non-significance was not attributable to statistical collinearity (VIF = 1.081, Tolerance = 0.925; Table 7). Rather, we interpret this as a confounding effect: in this sample, educational level (bachelor’s vs. master’s) was systematically correlated with age (median age: “Comprehensive Weakness Group” = 21 years vs. “Comprehensive Leadership Group” = 23 years). When educational level was included in the multivariable model, it likely captured the substantive developmental and training differences that age merely represented as a proxy variable. Consequently, age no longer contributed independent explanatory power beyond what educational background already accounted for. This pattern is consistent with classic confounding rather than multicollinearity: the two variables share a common causal relationship with SDL, but remain statistically independent. See Table 7 for multicollinearity diagnostics.

**Table 5:** Parallel lines test

Model	Chi-Square	df	Sig.
Null Hypothesis			
General	5.391	7	0.612

**Table 6:** Results of ordered logistic regression analysis

Variables	B	SE	Wald χ²	*P*	OR (95% CI)
Academic Burnout Scale	-0.153	0.017	83.157	**< 0.001**	0.858 (0.830 − 0.887)
Learning Motivation Scale	0.035	0.016	4.839	**0.028**	1.036 (1.004–1.068)
Age	-0.007	0.063	0.014	0.906	0.993 (0.878 − 1.122)
Current Enrollment [Ref: Master’s]	-2.117	0.31	46.667	**< 0.001**	0.120 (0.066 − 0.221)
Student council experience [Ref: No]	1.833	0.283	42.000	**< 0.001**	6.252 (3.592–10.877)
Career intent [Ref: No]	1.306	0.290	20.318	**< 0.001**	3.692 (2.092–6.514)
SDL training [Ref: No]	1.871	0.319	34.354	**< 0.001**	6.494 (3.473 − 12.147)

**Table 7:** Multicollinearity diagnostics

Variable (as in paper)	Tolerance	VIF
Current Enrollment (Bachelor’s vs. Master’s)	0.781	1.281
Experience as a student council or class committee member (Yes vs. No)	0.900	1.112
Interest in pursuing a career in nursing (Yes vs. No)	0.896	1.116
Experience with training related to self-directed learning (Yes vs. No)	0.903	1.108
Learning Motivation Scale (continuous)	0.873	1.146
Academic Burnout Scale (continuous)	0.797	1.255
Age (continuous)	0.925	1.081

Table 7 Note: Tolerance values are all > 0.1, and all VIFs are < 2, indicating no substantial multicollinearity

## Discussion

This study employed LPA to identify heterogeneous SDL competency profiles among post-2000s Chinese nursing students and examined factors associated with profile membership. Three profiles were identified—the “Comprehensive Weakness Group” (18.0%), the “Balanced Development Group” (48.0%), and the “Comprehensive Leadership Group” (34.0%)—revealing that SDL competencies in this population are distributed heterogeneously rather than uniformly. Despite an overall mean SDL score in the moderate range (65.29 ± 17.45), approximately one in five students exhibited low SDL across all dimensions, a subgroup that would have been obscured by variable-centered approaches reporting only mean-level estimates. This person-centered perspective challenges the conventional portrayal of nursing students’ SDL as “moderate overall” and demonstrates the educational value of LPA in detecting clinically and pedagogically meaningful subgroup variation.

### Current enrollment and self-directed learning skills

Bachelor’s students were significantly less likely than master’s students to belong to a higher SDL profile (OR = 0.120, 95% CI: 0.066–0.221), a pattern consistent with trends reported in prior research. Two mechanisms may help explain this association. First, graduate nursing education in China is structured around inquiry-based and research-driven tasks that require students to independently identify problems, synthesize evidence, and formulate solutions—a learning environment that may continuously reinforce SDL strategies and autonomy [32, 33]. Second, admission to Chinese graduate nursing programs requires passing highly competitive entrance examinations, which may preferentially select students with stronger pre-existing motivation and SDL capacity, thereby producing a “self-selection effect” within master’s cohorts. It should be noted, however, that the cross-sectional design of this study cannot establish a causal direction; it remains plausible that students who already possess higher SDL competencies are more likely to pursue graduate education rather than that graduate education itself produces higher SDL.

These observations carry practical implications for undergraduate nursing education in China. Educators may consider more systematically incorporating student-centered pedagogies—such as problem-based learning (PBL), flipped classroom approaches, and inquiry-based projects—into undergraduate curricula, as these methods may provide undergraduate students with structured opportunities to practice the self-direction, goal-setting, and self-monitoring skills that graduate-level curricula more naturally demand [33, 34]. 

It is also important to note that bachelor’s and master’s nursing programs in China differ not only in pedagogical approach but also in learning objectives, student characteristics, and institutional resources. Undergraduate programs primarily emphasize knowledge transmission and clinical skills acquisition, whereas master’s programs place greater weight on research competency and autonomous professional development. These systemic differences limit direct comparison and suggest that SDL interventions designed for undergraduates should be adapted to the specific structural context of undergraduate nursing education rather than simply replicating graduate-level approaches.

### Whether serving as a student council officer or class representative and the ability to study independently

Student leadership experience was positively associated with higher SDL profile membership (OR = 6.252, 95% CI: 3.592–10.877). This association is consistent with self-regulated learning theory [35], which posits that goal setting, strategy deployment, and self-monitoring are core components of both self-regulation and effective leadership. Students who serve in organizational roles must proactively plan activities, coordinate resources, manage timelines, and evaluate outcomes—activities that functionally parallel the key processes of SDL. Serving as a communication bridge between faculty and peers may further develop the interpersonal communication skills captured by the SDLI’s “interpersonal communication” dimension. These observations align with empirical findings from Park and Kim [36], who identified positive associations between self-leadership and SDL in nursing education. Importantly, the cross-sectional design precludes conclusions about directionality: it is equally possible that students who already possess stronger SDL competencies are more likely to seek or be selected for leadership roles. Regardless of causal direction, these findings suggest that creating structured leadership opportunities within nursing curricula—such as rotating group project leaders or peer teaching roles—may be associated with stronger SDL skill development [37]. Formal evaluation of such interventions through experimental designs would be a valuable next step.

### Whether to pursue a career in nursing and the ability to learn independently

Students who intended to pursue a nursing career were significantly more likely to belong to a higher SDL profile (OR = 3.692, 95% CI: 2.092–6.514). This association may be explained, in part, by career goal theory and self-determination theory: students with clear professional intentions may internalize the lifelong-learning demands of nursing practice at an earlier stage, motivating more proactive and self-sustaining learning behaviors [8, 9]. Accumulation of positive learning experiences may, in turn, reinforce self-efficacy, which has been widely linked to the adoption of deep learning strategies and persistence in the face of challenges [38]. However, given the cross-sectional design, the direction of this association remains uncertain; it is plausible that students with higher SDL competencies develop stronger professional commitment as a result of their more rewarding learning experiences, rather than the reverse.

From an educational standpoint, these findings suggest that fostering early professional identity development may be associated with stronger SDL motivation. Providing first-year students with structured early clinical observation opportunities may allow them to witness the professional value of nursing firsthand, potentially strengthening their commitment to the profession and stimulating intrinsic motivation for self-directed learning [39]. Longitudinal studies examining whether early career identity interventions prospectively predict SDL growth would help clarify the nature of this relationship.

### Impact of self-directed learning skills training on SDL competency development

Students with prior SDL training were substantially more likely to belong to a higher SDL profile (OR = 6.494, 95% CI: 3.473–12.147), representing one of the largest effect sizes in this study. This finding is consistent with the view that SDL is a learnable, trainable competency rather than a fixed dispositional trait. The four core SDL dimensions—learning motivation, planning and implementation, self-monitoring, and interpersonal communication—have been shown to be amenable to systematic instructional intervention [24]. A plausible mechanism involves positive reinforcement: students who acquire effective learning strategies may achieve better academic outcomes, which may strengthen self-efficacy and further consolidate SDL practices—a feedback loop described by Bandura [40]. Causal inference is nonetheless precluded by the cross-sectional design; students with higher baseline SDL may have been more likely to seek out or engage with SDL training, making the observed association partially attributable to self-selection.

These findings suggest that embedding structured SDL training modules into nursing curricula—rather than treating such training as an elective supplement—may be associated with meaningful gains in SDL competency. Practical examples include modular workshops on goal-setting techniques, learning plan construction, and self-monitoring strategies integrated across multiple years of the program. Rigorous evaluation through randomized or quasi-experimental designs would be needed to confirm the causal impact of such programs.

### Academic burnout and self-directed learning skills

Academic burnout was negatively associated with SDL profile membership (OR = 0.858, 95% CI: 0.830–0.887): the “Comprehensive Weakness Group” exhibited the highest burnout scores, while the “Comprehensive Leadership Group” demonstrated the lowest. This pattern is consistent with prior research linking academic burnout to diminished learning outcomes in nursing students. Conversely, poor SDL skills may compound burnout by reducing students’ ability to manage their learning workload effectively, potentially creating a self-reinforcing cycle. However, the cross-sectional design of this study does not permit determination of the direction of this relationship; a bidirectional, mutually reinforcing association between burnout and SDL is plausible and cannot be excluded. Longitudinal studies are needed to disentangle the temporal dynamics.

Regardless of causal direction, these findings suggest that early identification of students exhibiting high burnout—particularly those in the “Comprehensive Weakness Group”—combined with timely psychological support and burnout-reduction interventions, may represent a viable strategy for protecting SDL development. Systematic SDL skills training may therefore be associated with improvements in both self-management efficacy and perceived learning burden, though longitudinal evidence is needed to confirm these relationships [40]. 

### Motivation for learning and self-directed learning skills

Learning motivation was positively associated with higher SDL profile membership (OR = 1.036, 95% CI: 1.004–1.068), consistent with self-determination theory, which proposes that autonomous motivation supports the adoption of proactive self-directed learning strategies [41]. Students with stronger intrinsic motivation may maintain clearer academic and professional goals, providing an enduring drive for sustained self-directed learning. The small but statistically significant OR suggests that learning motivation may function more as a mediating or moderating variable—for example, partially explaining the effects of career intent and SDL training on profile membership—rather than as a strong independent predictor. This hypothesis warrants future investigation using structural equation modeling.

From a pedagogical perspective, career education and early clinical role modeling may serve as strategies for cultivating intrinsic motivation, as they help students connect learning activities to meaningful professional goals [42, 43]. 

### Implications for the future of nursing education

The most central educational implication of this study is that SDL heterogeneity requires educators to shift from uniform instructional approaches to stratified, profile-targeted interventions. The following profile-specific recommendations are proposed based on the study findings.

For the “Comprehensive Weakness Group” (18%): Early identification through SDL screening at program entry is recommended. Priority interventions should include: (1) structured SDL skill-building workshops covering goal-setting, learning planning, time management, and self-monitoring techniques; (2) one-to-one academic advising or mentoring to address individual learning barriers; (3) burnout screening and access to psychological support resources, including stress management and cognitive-behavioral skill-building programs; and (4) low-stakes peer collaboration activities to reduce anxiety and rebuild learning confidence.

For the “Balanced Development Group” (48%): Interventions for this majority group should focus on deepening SDL engagement and facilitating upward transition. Strategies may include: (1) rotating leadership roles in group projects and clinical simulations to provide structured practice in self-direction and planning; (2) career exploration workshops and early clinical observation to strengthen professional identity and intrinsic motivation; (3) incremental challenge through increasingly autonomous assignments that scaffold students toward independent inquiry; and (4) peer mentoring arrangements pairing “Balanced Development” students with members of the “Comprehensive Leadership Group.”

For the “Comprehensive Leadership Group” (34%): Strategies for this group should leverage their existing SDL strengths to benefit the broader cohort: (1) formalized peer mentoring and teaching roles (e.g., facilitating study groups, demonstrating learning strategies); (2) advanced research participation, honors projects, or clinical scholarship opportunities that sustain high-level SDL engagement; and (3) recognition mechanisms to reinforce and model exemplary SDL behaviors for peers.

At the institutional level, SDL competency training should be systematically embedded in the nursing curriculum as a longitudinal thread across all program years, supported by dynamic assessment and feedback mechanisms that allow ongoing monitoring and targeted adjustment of interventions.

### Limitations

This study has several limitations that should be considered when interpreting the findings. First, the sample was recruited from two medical universities in Liaoning and Shandong provinces using convenience sampling. Although these institutions offer both bachelor’s and master’s nursing programs representative of northeastern and eastern Chinese nursing education, the findings may not generalize to other geographic regions, institutions with different educational resources, or nursing programs in countries with different educational systems. In particular, nursing education structures vary substantially across countries: curricula in North America, Europe, and Australia typically place greater emphasis on student-centered, active learning methodologies from early program years, and SDL profiles identified in this sample may differ under those educational conditions. Readers should exercise caution in applying these findings beyond the Chinese higher nursing education context. Second, the concurrent inclusion of both bachelor’s and master’s students—two groups that differ markedly in program structure, learning demands, and student developmental stage—should be noted as a potential source of heterogeneity in the sample. The educational level variable captured some of this variation in the regression model, but unmeasured between-level differences (e.g., faculty mentoring culture, research involvement, curriculum design) may have influenced the profiles in ways not fully accounted for. Third, all SDL competency data were collected via self-report scales, which may introduce social desirability bias and may not fully reflect objective SDL behaviors. Fourth, the cross-sectional design precludes causal inference regarding any of the identified associations; the direction of relationships between SDL profiles, burnout, motivation, and experiential factors cannot be determined from this study alone. Future research should adopt multicenter, longitudinal designs, incorporate objective behavioral indicators of SDL (e.g., academic performance, library and resource usage), and examine whether the three identified profiles remain stable over time or evolve across students’ educational trajectories. Comparative studies across different national educational systems would further advance understanding of how institutional and cultural contexts shape SDL heterogeneity.

## Conclusion

This study applied LPA to identify three heterogeneous SDL competency profiles among post-2000s Chinese nursing students: the “Comprehensive Weakness Group” (18.0%), the “Balanced Development Group” (48.0%), and the “Comprehensive Leadership Group” (34.0%). This person-centered approach revealed that SDL competencies are not uniformly distributed at a “moderate overall level,” but rather exhibit meaningful within-population heterogeneity that aggregate mean scores cannot capture. Educational level, student leadership experience, nursing career intent, SDL training experience, academic burnout, and learning motivation were all independently associated with SDL profile membership. Nursing educators are encouraged to implement differentiated, profile-specific interventions: structured SDL training and psychological support for the “Comprehensive Weakness Group”; empowerment and leadership opportunities for the “Balanced Development Group”; and formalized peer mentoring roles for the “Comprehensive Leadership Group.” A multi-tiered institutional approach—embedding SDL training systematically into curricula and monitoring competency development longitudinally—offers the most promising pathway for enhancing SDL across the entire post-2000s nursing student population.

## Data Availability

The datasets generated and/or analysed during the current study are not publicly available due to the protection of participants’ privacy and confidentiality, but are available from the corresponding author on reasonable request.

## Abbreviations

aBIC: Sample-size-adjusted Bayesian Information Criterion
AIC: Akaike Information Criterion
BIC: Bayesian Information Criterion
BLRT: Bootstrap Likelihood Ratio Test
CI: Confidence Interval
GPA: Grade Point Average
LMR: Lo-Mendell-Rubin Test
LPA: Latent Profile Analysis
OR: Odds Ratio
PBL: Problem‑Based Learning
SD: Standard Deviation
SDL: Self‑Directed Learning
SDLI: Self‑Directed Learning Ability Inventory
